# Fluticasone furoate/Vilanterol 92/22 μg once-a-day vs Beclomethasone dipropionate/Formoterol 100/6 μg b.I.D.: a 12-month comparison of outcomes in mild-to-moderate asthma

**DOI:** 10.1186/s40248-018-0131-x

**Published:** 2018-06-15

**Authors:** Roberto W. Dal Negro, Luca Bonadiman, Paola Turco

**Affiliations:** 1 National Centre for Respiratory Pharmacoeconomics and Pharmacoepidemiology, Verona, Italy; 2 Research & Clinical Governance, Verona, Italy

**Keywords:** Adherence to treatment, Beclomethasone dipropionate/Formoterol, Clinical outcomes, Fluticasone furoate/Vilanterol, Mild-to-moderate asthma, Time dependency, Twelve-month survey

## Abstract

**Background:**

Bronchial asthma is an inflammatory disease of the airways. Beclomethasone dipropionate/Formoterol (BDP/F) and Fluticasone furoate/Vilanterol (FF/V) are two of the most effective LABA/ICS combinations for managing persistent bronchial asthma. Aim of the study was to compare the outcomes achieved in mild-to-moderate asthma patients assuming BDP/F 100/6 μg b.i.d. (Group A) or FF/V 92/22 μg once-daily (Group B) for 12-months. No head-to-head long-term comparison is available at present.

**Methods:**

Data were automatically and anonymously obtained from the institutional database: FEV_1_% predicted values; the exacerbation and hospitalization rates; days of hospitalization; GP and/or specialist visits; days of inactivity; courses of systemic steroids and/or antibiotics were recorded at baseline and after 3, 6 and 12 months of both treatments. The overall adherence to treatments was also calculated. The propensity score method was used for matching and comparing the two cohorts of patients; Anova and Wilcoxon tests were used for checking the trends and time-to-time comparisons over the period; statistical significance was accepted for *p* < 0.05.

**Results:**

The PS-matching process returned a cohort of 40 group A patients matched with 40 patients of group B, fully comparable for demographics, clinical characteristics, and comorbidities. The improvement in lung function was significant in both groups (*p* < 0.001), even if it was significantly higher and time-dependent in group B. The mean (±SE) exacerbation rate/patient changed from 0.63 (±0.13) at baseline to 0.53 (±0.12) after three; to 0.58 (±0.13) after six, and to 0.60 (±0.18) after twelve months in group A (p = ns), while from of 1.05 (±0.16) at baseline, to 0.28 (±0.07) after three; to 0.33 (±0.08) after six, and to 0.18 (±0.08) after twelve months in group B (*p* < 0.001), respectively. The mean hospitalization rate/patient changed from 0.25 ± 0.07 at baseline to 0.15 (±0.06) after three; to 0.08 (±0.04) after six, and to 0.13 (±0.05) after twelve months in group A (p = ns), while from 0.30 (±0.07) at baseline to 0.08 (±0.04) after three; to 0.10 (±0.05) after six, and to 0.03 (±0.03) after twelve months in group B (*p* < 0.001), respectively. Also mean duration of hospitalization and days of inactivity were in favour of FF/V treatment over time (in both cases *p* < 0.001). GP’s visits were reduced by both treatments (*p* < 0.007 in group A and p < 0.001 in group B, respectively, while Specialist’s visits only dropped during FF/V (*p* < 0.001). Steroid and antibiotic courses were significantly reduced by both treatments, even if more systematically in group B (p < 0.001 vs *p* < 0.007, and p < 0.001 vs *p* < 0.044, respectively). Moreover, changes in all outcomes considered proved time-dependent during the FF/V treatment only, particularly over the second semester. Finally, the overtime adherence to treatment was higher by 22 days during FF/V .

**Conclusions:**

Both the ICS/LABA combinations proved effective, even if characterized by different patterns of effectiveness either in terms of lung function and of long-term clinical outcomes. Only the once-daily inhalation of combined FF/V 92/22 μg once-daily optimized systematically the exacerbation and hospitalization rates in mild-to-moderate asthma, together with all other outcomes over time. The effectiveness of FF/V 92/22 once-daily μg proved progressive and time-dependent over the twelve-month period of the study, and associated to a higher adherence to treatment.

## Background

Bronchial asthma is a chronic inflammatory disease of the airways which is characterized by airflow limitation, usually reversible spontaneously or following therapy, bronchial hyper-responsiveness and accelerated decline in lung function, and the occurrence of exacerbations [1].

The excessive presence and activation of inflammatory cells within the mucosal, muscular and vascular structures of the airways are the underlying mechanisms responsible for asthma, which cause the release of inflammation mediators and the remodeling of the airways. Clinical manifestations of asthma consist of recurrence of cough, dyspnea, wheezing (at rest and/or by physical exertion), and chest tightness [1]. These manifestations can change among individuals and/or in the same subject over time [2].

According to WHO estimates, 235 million people suffer from asthma. The Italian National Institute of Statistics (ISTAT) survey on health and use of health services estimated a prevalence of asthma of 4.2% (female 4.3%, male 4.2%) in Italy in 2012 [3], and the total burden of asthma was estimated in about 5 billion euro per year in Italy [4].

Severity of the disease is evaluated on frequency of symptoms, value of forced expiratory volume in 1 s (FEV_1_), variability of peak expiratory flow (PEF), reversibility of airway obstruction, exacerbation rate, quality of life. Four levels of asthma severity are recognised: mild intermittent, mild persistent, moderate persistent and severe persistent.

Asthma cannot be cured, but appropriate management may control the disorder and enable people to enjoy a good quality of life [2]. The main goal of asthma therapy is to achieve and maintain the control of the disease in real life.

The therapeutic strategy includes two main categories of drugs: the controller medications which must be assumed regularly to keep the disease under control, and the rescue medications which relieve the acute bronchoconstriction and related symptoms. Since asthma is an inflammatory disease, inhaled corticosteroids (ICS) are the most effective controller medications currently available and represent the first choice of treatment, to which long-acting beta_2_-agonists bronchodilators (LABA) can be added. The combination of these two categories of drugs is the recommended therapeutic strategy for persistent asthma [1].

Two of the most recent LABA/ICS combinations for persistent bronchial asthma are the Fluticasone furoate/Vilanterol (FF/V) 92/22 μg delivered via the Ellipta device [5–7] and the Beclomethasone dipropionate/Formoterol 100/6 μg (BDP/F) delivered via the Nexthaler device [8–10]. While the former combination covers twenty-four hours and is assumed at an once-a-day regimen, the latter has to be assumed twice daily (bis in die, b.i.d.).

Although several studies investigated both the effectiveness and the safety of these two ICS/LABA combinations singularly, no long-term comparison is still available to our knowledge.

## Aim

The aim of the present study was to estimate and compare the outcomes achievable by mild-to-moderate asthma patients assuming BDP/F 100/6 μg b.i.d. to those of patients assuming FF/V 92/22 μg once-a-day over a twelve-month treatment.

## Methods

The study was an observational, retrospective analysis on asthmatic patients referring over the period February–September 2015 to the Lung Unit of the Specialist Medical Centre (CEMS), Verona, Italy.

Data were obtained automatically and anonymously from the institutional, UNI EN ISO 9001–2008 validated database, and the classic Boolean algebraic formula were used for selections [11]. Selection criteria were: mild-to-moderate asthma subjects of both genders; > 18 years of age; non-smoker; with a normal cognitive function; in a stable respiratory condition (spirometrically assessed) in the last 2 weeks before the study start; assuming BDP/F 100/6 μg b.i.d (Group A) or FF/V 92/22 μg once-a-day (Group B) for 12 (±2) months. At baseline sex, age, the absolute and the % predicted values of forced expiratory volume in 1 s (FEV_1_ in Litres and FEV_1_ as % predicted), and comorbidities of the patients were recorded. All patients were followed for 12 (±2) months. FEV_1_ values; number of relapses and of related hospitalizations; duration of hospitalization (in days); number of general practitioner (GP) and/or specialist visits; days of inactivity; and number of courses of systemic steroids and antibiotics were recorded over the study period at baseline and after 3, 6, and 12 months of both treatments. Baseline values for outcomes were corresponding to values assessed over the three months preceding the index date for selections. Furthermore, as both the inhaler devices used are provided with a precise dose counter, the patients’ adherence to both treatments was also recorded monthly since the index data (via monthly telephone calls and registration of the remaining doses in the device), and expressed in % inhalations vs the expected number of inhalations at each time of the study.

In order to compare the outcomes achieved in the two groups of patients, the propensity score matching method (PS) [12] was used in STATA [13]. The propensity score matching method summarizes pretreatment characteristics of each subject into a single-index variable (the propensity score) that makes the matching feasible. In this study a logit regression to estimate the propensity score on the baseline covariates age, sex, FEV_1_ (%) and presence of comorbidities, was used. Moreover, the propensity score matching was performed without replacement, i.e. each of 40 patients of the Group B was matched with only one patient of the Group A.

Data reported at baseline and after three months of both treatments correspond to those already published in a previous study which was limited to a twelve-week observational period on the same cohort of patients [14]. Data collected from the same patients’ cohort after 6 and 12 months were implemented in the present study in order to complete a four-point trend over 12 months of both treatments.

The analysis of variance was used to check the four-point trend (such as: baseline; at 3, 6, and 12 months) recorded in each treatment group for all outcomes. Finally, the extent of changes achieved in both treatment groups by each outcome considered was also compared at the same times by Wilcoxon test. Statistical significance was accepted for *p* < 0.05.

The study was approved by the R&CG Ethical Committee during the session officially held on January 11^th^, 2016. The patients’ consent to participate was not inserted because data were obtained automatically and anonymously.

## Results

Clinical data of 77 patients treated with BDP/F 100/6 μg b.i.d (Group A) and of 40 patients treated with FF/V 92/22 μg once-a-day (Group B) were obtained. Characteristics of the entire cohort and of the PS-matched cohort at baseline are summarized in Table 1. At baseline, male prevalence was 33.8% in group A and 37.5% in group B. Mean (±SE) age was 51.9 (±1.60) in group A, and 50.2 (±2.43) in group B. Mean (±SE) FEV_1_ in litres (L) was 2.4 (±0.09) in group A, and 2.5 ((±0.12) in group B. Mean (±SE) FEV_1_% pred. was 82.2% (±1.14) and 81.9% (±2.00) in group A and B, respectively. Patients with perennial allergy were 61.0% (47/77) in group A, and 62.5% (25/40) in group B, while those with seasonal allergy were 39.0% (30/77) in group A, and 37.5% (15/40) in group B, respectively. The percentage of patients with established comorbidities was 37.7% in group A, and 42.5% in group B. The following comorbid diseases were equally reported in both groups: arterial hypertension, kyphoscoliosis, obesity, severe depression, AIDS, diabetes mellitus, severe osteoporosis, and ischemic heart disease. In particular, arterial hypertension was the most prevalent comorbidity in both groups: 12.5% in group A, and 10.4% in group B, respectively.

**Table 1:** Characteristics of the entire cohort and of the PS-matched cohort at baseline

	Overall cohort			PS-matched cohort		
	Group A	Group B	Difference Group B – Group A	Group A	Group B	Difference Group B – Group A
n	77	40		40	40	
Males (n) (%)	26 (33.8%)	15 (37.5%)	−11 (−3.80%)	15 (37.5%)	15 (37.5%)	0
Mean Age (years) (±s.e.)	51.87 (±1.60)	50.2 (±2.43)	−1.69	49.40 (±2.05)	50.2 (±2.43)	0.78
Mean FEV_1_% predicted (±s.e.)	82.2 (±1.14)	81.9 (±2.00)	−0.30	82.40 (±1.6)	81.9 (±2.00)	−0.47
Comorbidities (% of patients)	37.7%	42.5%	−4.80%	42.5%	42.5%	0%

Forced expiratory volume in 1 s, predicted values (FEV_1_%)

Group A: patients treated with Beclomethasone dipropionate/Formoterol 100/6 μg b.i.d

Group B: patients treated with Fluticasone furoate/Vilanterol 92/22 μg once-a-day

The PS-matching process, designed as matching on the baseline covariates, gender, age, FEV_1_ and comorbidities, returned a cohort of 40 group A patients of the entire cohort matched with 40 patients of group B. The demographics and clinical characteristics of the PS-matched cohort at the baseline are described in Table 1. The male prevalence in group A was the same as in group B (37.5%). Mean age (±SE) was 49.4 (±2.05) in group A and 50.2 (±2.43) in group B, respectively. Mean (±SE) FEV_1_% pred. was 82.4% (±1.63) in group A and 81.9% (±2.00) in group B. The presence of comorbidities was balanced (42.5%) in both groups (Table 1).

**Fig. 1:**
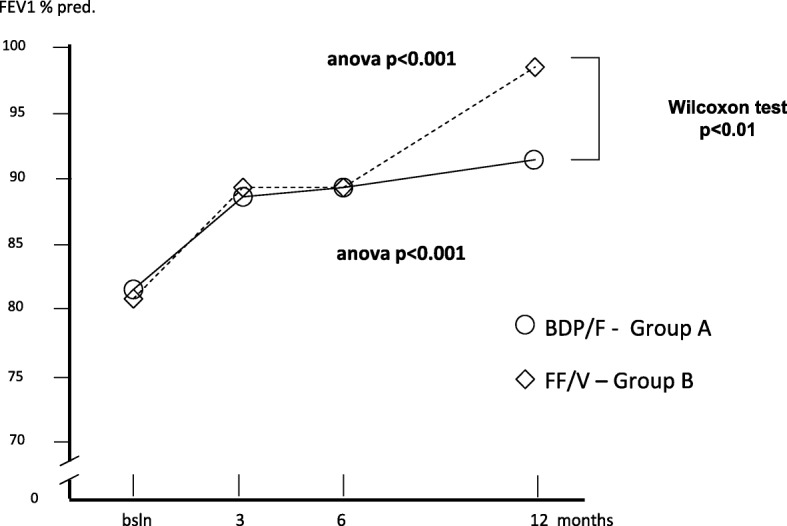
Changes in mean FEV_1_% pred. over 12 months

Table 2 summarizes all changes calculated for each variable over the study period in each treatment group. Mean FEV_1_% pred. changed from 82.40% (±1.63) at baseline to 87.08% (±1.58) after 3 months; to 89.98% after 6 months, and to 91.88% after 12 months (Anova = *p* < 0.001) in group A, while in group B, mean (±SE) FEV_1_% pred. changed from 81.93% (±2.00) at baseline, to 89.50% after 3, to 90.9% after 6, and to 99.1% after 12 months of treatment (Anova = *p* > 0.001). Even if the overall trends of lung function proved significantly improved with both treatments, treatment B induced a FEV_1_% pred. improvement in the second semester of the study which was significantly higher than that obtained with treatment A (t test: *p* < 0.01) (Fig. 1).

**Table 2:** Means ± s.e. for each variable calculated in the two groups of patients at baseline, and at 3, 6, and 12 months of treatments (*n* = 40 + 40 matched)

	Group A (means ± s.e.)				Group B (means ± s.e.)			
	T0	T3	T6	T12	T0	T3	T6	T12
FEV_1_% pred.	82.40 ± 1.69	87.08 ± 1.58	89.98 ± 2.0	91.88 ± 2.13	81.93 ± 2.0	89.50 ± 2.64	90.9 ± 3.23	99.1 ± 3.24
Exac. Rate/p	0.63 ± 0.13	0.53 ± 0.12	0.58 ± 0.13	0.60 ± 0.18	1.05 ± 0.16	0.28 ± 0.07	0.33 ± 0.08	0.18 ± 0.08
Hosp. rate/p.	0.25 ± 0.07	0.15 ±0.06	0.08 ± 0.04	0.13 ± 0.05	0.30 ± 0.07	0.08 ± 0.04	0.10 ± 0.05	0.03 ± 0.03
H. duration (days)	0.83 ± 0.26	0.28 ± 0.12	0.48 ± 0.38	0.60 ± 0.31	0.88 ± 0.31	0.08 ± 0.04	0.10 ± 0.05	0.03 ± 0.03
Inactivity (days)/p	2.88 ± 0.63	1.53 ± 0.27	1.10 ± 0.21	1.45 ± 0.58	3.35 ± 0.63	0.60 ± 0.19	1.35 ± 0.34	0.83 ± 0.39
Spec. Visits/p	0.68 ± 0.13	0.68 ± 0.11	0.65 ± 0.08	1.05 ± 0.13	0.70 ± 0.16	0.28 ± 0.07	0.35 ± 0.08	0.23 ± 0.08
GP visits/p	1.33 ± 0.23	0.85 ± 0.15	0.55 ± 0.13	0.63 ± 0.15	1.53 ± 0.24	0.38 ± 0.12	0.78 ± 0.12	0.25 ± 0.09
Courses of steroids/p	0.90 ± 0.15	0.73 0.13	0.48 ± 0.11	0.30 ± 0.09	1.08 ± 0.16	0.33 0.08	0.30 ± 0.08	0.13 ± 0.06
Courses of antibiotics/p.	0.85 ± 0.16	0.63 0.10	0.43 ± 0.12	0.40 ± 0.11	1.03 ± 0.13	0.35 0.08	0.33 ± 0.08	0.15 ± 0.08

Group A: Beclomethasone dipropionate/Formoterol (BDP/F) 100/6 μg b.i.d., via Nexthaler. Group B: Fluticasone furoate/Vilanterol (FF/V) 92/22 μg o.d., via Ellipta

**Fig. 2:**
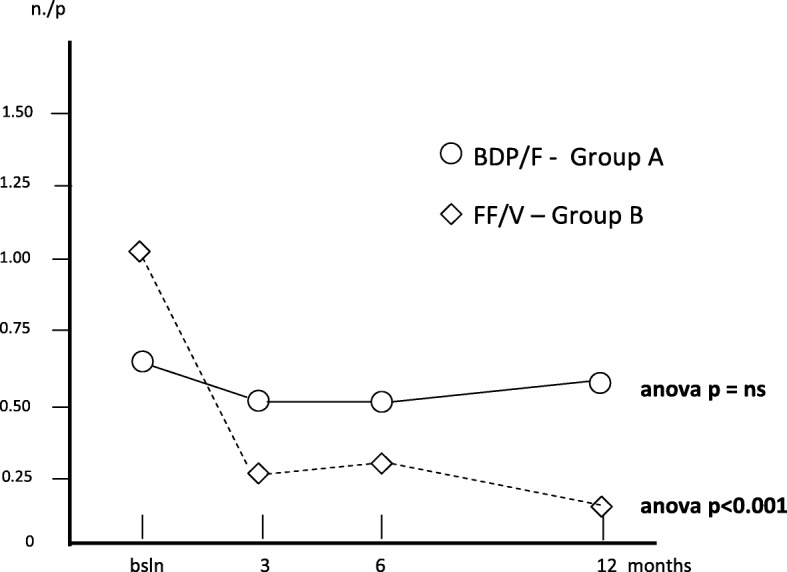
Changes in mean n. exacerbations/p. over 12 months

The mean (±SE) exacerbation rate per patient was 0.63 (0.13) at baseline; 0.53 (±0.12) after 3; 0.58 (0.13) after 6, and 0.60 (0.18) after 12 months in group A (Anova: p = ns), while the corresponding rate in group B was 1.05 (0.16) at baseline; 0.28 (0.07) after 3; 0.33 (0.08) after 6, and 0.18 (0.08) after 12 months of treatment (Anova: *p* < 0.001) in group B, respectively. In this case, the reduction of the rate achieved with treatment B was substantial and significant over the entire study period (Fig. 2).

**Fig. 3:**
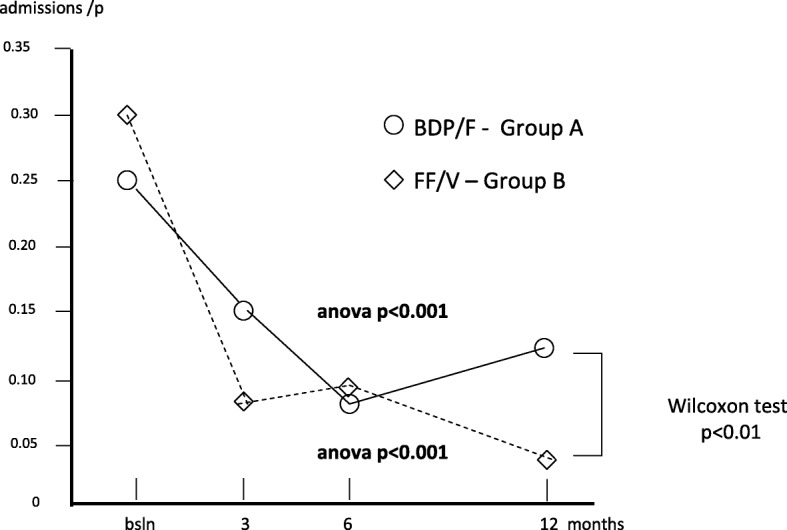
Changes in mean n. of hospitalizations/p. over 12 months

The average (±SE) rate of asthma-induced hospitalizations per patient was 0.25 (0.07) at baseline; 0.15 (±0.06) after 3; 0.08 (0.04) after 6, and 0.13 (0.05) after 12 months in group A (Anova: p = ns), while the corresponding mean number in group B was 0.30 (0.07) at baseline; 0.08 (0.04) after 3; 0.10 (0.05) after 6, and 0.03 (0.03) after 12 months (Anova; *p* < 0.001), respectively. The difference was statistically significant in favour of group B (t paired test: *p* < 0.04) over the second semester (Fig. 3).

Also in this case, even if the overall trends of the hospitalization rates proved significantly improved with both treatments, the reduction obtained in the second semester of treatment B was really substantial and significantly higher (t test: *p* < 0.01) (Fig. 3).

**Fig. 4:**
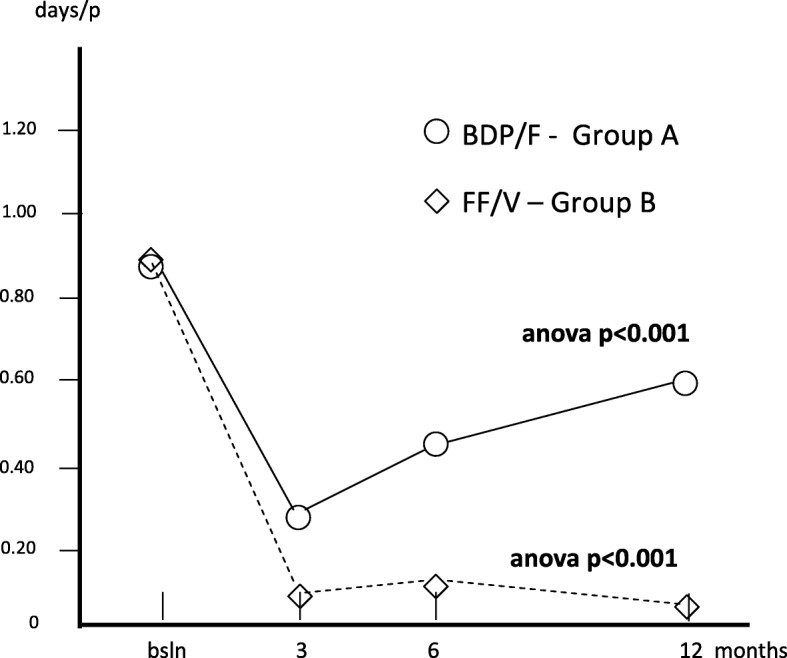
Changes in mean duration of hospitalization/p. over 12 months. s

The corresponding mean duration of hospitalization was 0.83 (0.26) at baseline; 0.28 (0.12) after 3; 0.48 (0.38) after 6, and 0.60 days (0.31) after 12 months (Anova: p = ns) in group A, while the corresponding mean duration in group B was 0.88 (0.31) at baseline; 0.08 (0.04) after 3; 0.10 (0.05) after 6, and 0.03 days (0.03) after 12 months (Anova: *p* < 0.001), respectively. The difference in favour of treatment B was extremely clear over the second semester of treatment (Fig. 4).

**Fig. 5:**
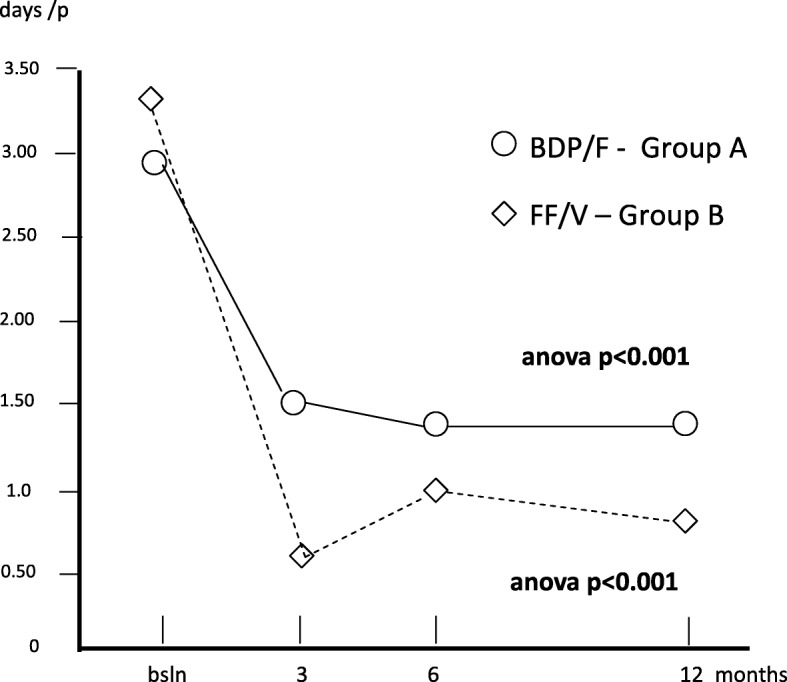
Changes in mean duration of inactivity/p. over 12 months

The mean duration of inactivity was 2.88 (0.63) at baseline; 1.53 (0.27) after 3; 1.40 (0.27) after 6, and 1.45 days (0.58) after twelve months (Anova: *p* = 0.11) in group A, while the corresponding duration in group B was 3.35 (0.63) at baseline; 0.60 (0.19) after 3; 1.10 (0.21) after 6, and 0.83 days (0.39) after 21 months (Anova; *p* < 0.001), respectively. The difference was statistically significant in favour of group B over the entire period of treatment (Fig. 5).

**Fig. 6:**
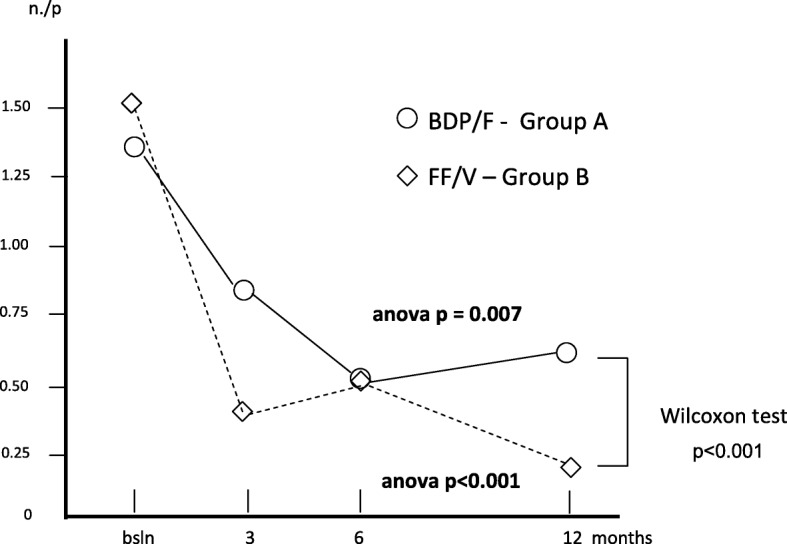
Changes in mean n. GP visits/p. over 12 months

The mean number of GP visits per patient was 1.33 (0.23) at baseline; 0.85 (0.15) after 3; 0.55 (0.13) after 6, and 0.63 (0.15) after 12 months (Anova; *p* < 0.007) in group A, while the corresponding number in group B was 1.53 (0.24) at baseline; 0.38 (0.12) after 3; 0.55 (0.09) after 6, and 0.25 (0.06) after 12 months (Anova; *p* < 0.001), respectively. The frequency of GPs’ visits was significantly lower during the second semester of treatment B (t test: *p* < 0.01) (Fig. 6).

**Fig. 7:**
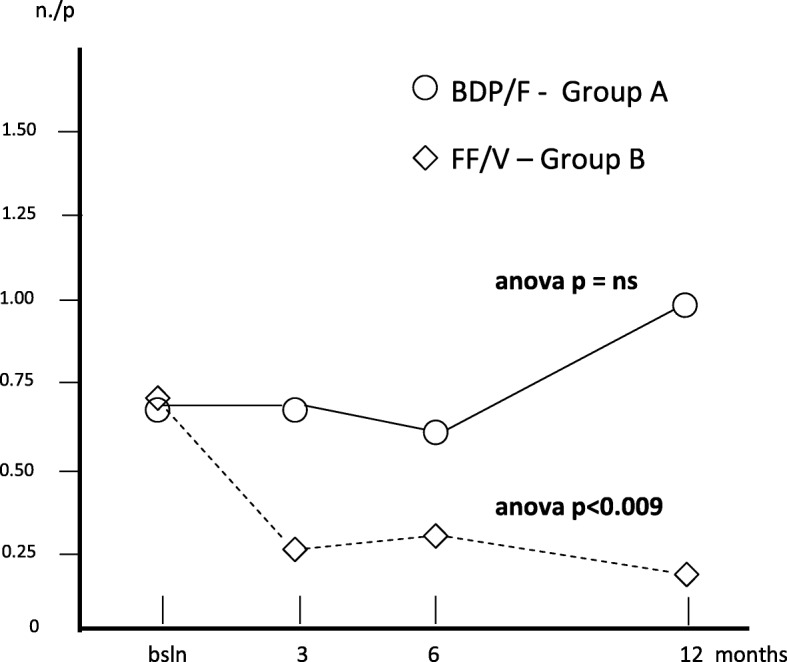
Changes in mean n. Specialist visits/p. over 12 months

The mean number of Specialist visits per patient was 0.68 (0.13) at baseline; 0.68 (0.11) after 3; 0.65 (0.08) after 6, and 1.05 (0.13) after 12 months (Anova: p = ns), while the corresponding number in group B was 0.70 (0.16) at baseline; 0.28 (0.07) after 3; 0.35 (0.08) after 6, and 0.23 (0.08) after 12 months (Anova: *p* < 0.009), respectively. The difference was in favour of group B for all the three times of follow up (t paired test: *p* < 0.002; *p* < 0.005, and *p* < 0.001, respectively). The difference between the treatments proved highly significant in favour of group B over the entire study period (Fig. 7).

**Fig. 8:**
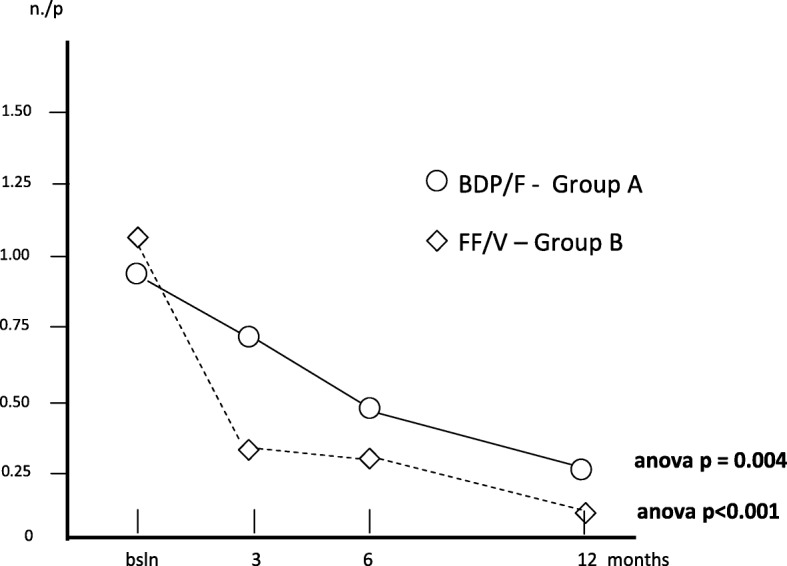
Changes in mean courses of oral steroids/p. over 12 months

The mean number of courses of systemic steroids per patient was 0.90 (0.15) at baseline; 0.73 (0.13) after 3; 0.48 (0.11) after 6, and 0.30 (0.09) after 12 months (Anova: *p* < 0.004) in group A, while the corresponding number in group B was 1.08 (0.16) at baseline; 0.33 (0.08) after 3; 0.30 (0.08) after 6, and 0.13 (0.08) after 12 months (Anova: *p* < 0.001), respectively (Fig. 8).

**Fig. 9:**
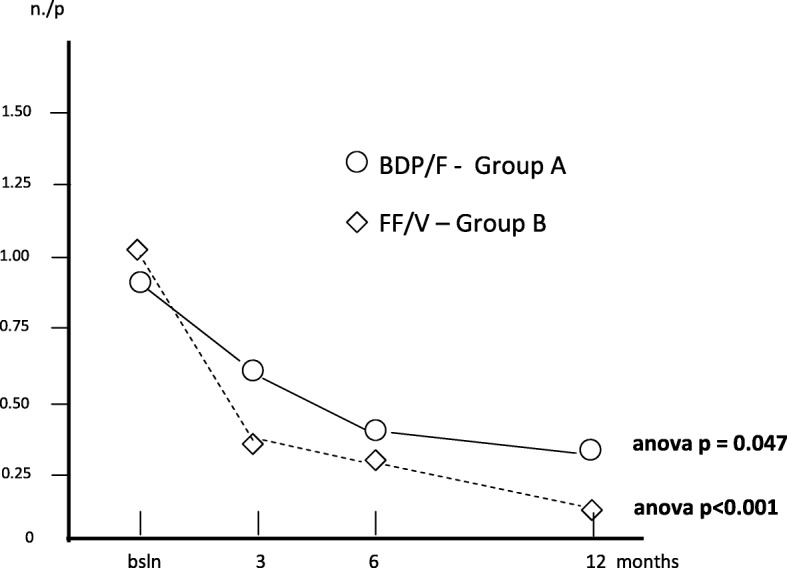
Changes in mean courses of antibiotics/p. over 12 months

Finally, the mean number of courses of antibiotics per patient was 0.85 (0.16) at baseline; 0.63 (1.10) after 3; 0.43 (0.12) after 6, and 0.40 (0.11) after 12 months (Anova: 0.047), while the corresponding number in group B was 1.03 (0.13) at baseline; 0.35 (0.08) after 3; 0.33 (0.08) after 6, and 0.15 (0.08) after 12 months (Anova: p < 0.001), respectively (Fig. 9).

The adherence to prescribed treatments calculated in terms of expected doses over the period (stemming from the index date) was of 82.2% at three; 81.7% at six, and 80.8 at twelve months in group A, while of 93.3% at three; 91.7 at six, and 90.6 at twelve months in group B, respectively. In other words, an average of 132 doses (approximately corresponding to 66 days of treatment) in group A, and to 44 doses were skipped (corresponding to 44 days of treatment) in group B.

No relevant side effect was reported in both groups of patients. Transient hoarseness was recorded in 5 patients in group B and in three patients of group A, while transient tachycardia was recorded in two patients of group A and in one patient in group B.

## Discussion

A variable degree of airway obstruction related to a variable extent of underlying airway inflammation usually characterizes bronchial asthma. In persistent mild-to-moderate asthma a therapeutic strategy based on the regular assumption of ICS, or ICS/LABA is recommended in order to prevent and/or avoid the occurrence of asthma exacerbations.

Results of different treatments can be affected by several factors, such as: the pharmacological peculiarities of the molecules prescribed; the daily regimen (namely the frequency of inhalations required for a twenty-four-hour efficacy); the usability of inhaler devices adopted for the drug(s) delivery; the patient’s adherence to treatment; the existence of comorbidities; the cost of treatment, and the indices considered in the study (such as, lung function only, rather than clinical outcomes).

The present observational, retrospective, matched study, aimed to compare outcomes achievable in mild-to-moderate asthma patients assuming FF/V once-daily or BDP/F for 12-months, represents the very first head-to-head comparison between these two LABA/ICS combinations in asthma to our knowledge, and here clinical outcomes are assessed over a long-term period.

Actually, in a previous pharmaco-economic study, a short-term cost-analysis carried out over twelve weeks suggested the superiority of FF/V 99/22 once-daily via Ellipta when compared to DP/F b.i.d. via Nexthaler [14]. This superiority in mild-to-moderate asthma was related to a significant higher improvement in lung function together with a significant reduction of GP’ and Specialist’s visits, and of extra-medication, thus indirectly confirming a better control of asthma in daily life. It was also observed a 50% drop in hospitalization cost in the same study, even if this tendency did not reach the statistical significance due to the dispersion of data occurring during the too limited period of investigation [14].

In terms of lung function, both treatments confirmed effective in improving FEV_1_% predicted significantly also in the present study. The net improvement achieved in group B proved once again significantly higher, but also progressive, according to a time-dependent trend, particularly over the second six months of treatment.

A novel evidence came out from the present study: beyond lung function, all main clinical outcomes proved clearly in favour of FF/V once-daily when compared to BDP/F b.i.d. Actually, the long-term treatment likely contributed to enhance and magnify the extent of FF/V clinical convenience, previously only suggested during a short-term therapeutic strategy [14]. In particular, the dramatic reduction of exacerbation and hospitalization rates, patients’ duration of inactivity; the frequency of referral to the GP and the Specialist, and the number of courses of oral steroids and antibiotics represents a crucial confirmation of the much substantial and more effective asthma control achievable with long-term FF/V once-daily in real-life.

Even if the two compared ICS/LABA combinations are active and regarded as equally effective in persistent asthma [5–10], nonetheless present data emphasize that they are characterized by a different profile concerning their long-term clinical efficacy in mild-to-moderate asthma. Actually, the systematic trend of a progressive, time-dependent improvement of all main clinical outcomes highlights how FF/V once-daily should be regarded as the much more convenient strategy for longer lasting treatments.

On the other hand, Formoterol and Vilanterol as well as Beclomethasone dipropionate and Fluticasone furoate are characterized by different pharmacokinetics and pharmacodinamics [15–17]. The corresponding fixed combinations obviously reflect these pharmacological patterns which support and provide different aspects of their clinical efficacy and effectiveness also in clinical terms. In particular, the higher selectivity and persistency on steroid receptors in favour of Fluticasone furoate, together with the higher selectivity and persistency on ß_2_- receptors in favour of Vilanterol represent crucial aspects from this point of view [15–17]. Actually, differently than in the case of Formoterol and Beclomethasone dipropionate which require twice-daily administration [18], these are the peculiarities which allow the long-lasting therapeutic action of the FF/V combination.

The once-daily assumption has been supposed to foster the patients’ adherence during long-term therapeutic strategies [7, 19, 20]. The substantial difference between treatments in terms of number of skipped doses (which corresponds to skipping days of treatment) over the twelve months observational period as assessed in the present study is strongly supporting this hypothesis. In other words, when compared to BDP/F b.i.d., FF/V once-daily allowed a longer adherence by 22 days in real life, which likely contributes *per sé* to explain the better and the time-dependent asthma control achievable with this treatment. To note that this result should be regarded as independent of the inhaler devices used by patients (namely, Nexthaler and Ellipta) as both characterized by a quite similar handling and an equal number of steps needed for inhalation actuation [21, 22].

Finally, hospitalization and exacerbation rates, as well as patients’ absenteeism and medical referrals represent the main components of asthma annual costs [20–22]. The dramatic and progressive drop in these four indices obtained over the twelve-month treatment with FF/V strongly supports and emphasizes the economic convenience of this strategy when compared to that of BDP/F for the long-term management of mild-to-moderate asthma.

The present study has some limits. One is represented by the relative small number of subjects included, even if well matched by means of the propensity score. Moreover, the study consists in a mono-centric investigation, even though patients were from all Italian regions, anonymously selected. On the other hand, some points of strength just consist in the automatic selection of subjects from a unique database, associated to the use of the propensity score matching method which assures a strictly objective system for comparison between the two subjects’ samples. Finally, the patients’ adherence to treatments was not calculated according to the usual criteria adopted during usual clinical trials. Anyway, the registration of the number of doses monthly remaining in both devices (each provided with a precise dose-counter) rendered information collected in real life pretty reliable and acceptable.

## Conclusions

The present study showed that the once-daily inhalation of combined Fluticasone furoate/Vilanterol 92/22 μg once-daily for twelve months consents an enhanced and time-dependent efficacy in terms of lung function and of all main clinical outcomes when compared to BDP/F 100/6 μg b.i.d. in mild-to-moderate asthma. Stemming from the extent of the systematic improvement of clinical outcomes achieved over the FF/V 92/22 μg treatment, the corresponding long-term economic consequences are easily and quantitatively presumable.
