# Correction to: Fluticasone furoate/Vilanterol 92/22 μg once-a-day vs Beclomethasone dipropionate/Formoterol 100/6 μg b.I.D.: a 12-month comparison of outcomes in mild-to-moderate asthma

**DOI:** 10.1186/s40248-018-0144-5

**Published:** 2018-07-24

**Authors:** Roberto W. Dal Negro, Luca Bonadiman, Paola Turco

**Affiliations:** 1 National Centre for Respiratory Pharmacoeconomics and Pharmacoepidemiology, Verona, Italy; 2 Research & Clinical Governance, Verona, Italy

## Correction to: Multidisciplinary Respiratory Medicine (2018) 13:18 DOI: 10.1186/s40248-018-0131-x

After publication of the Original research article [1] it was brought to our attention that the sentence at pag 6 (between Fig. 3 and Fig. 4)) must be corrected as follows: “The mean duration of inactivity was 2.88 (0.63) at baseline; 1.53 (0.27) after 3; 1.40 (0.27) after 6, and 1.45 days (0.58) after twelve months (Anova: p = 0.11) in group A, while the corresponding duration in group B was 3.35 (0.63) at baseline; 0.60 (0.19) after 3; 1.10 (0.21) after 6, and 0.83 days (0.39) after **12** months (Anova; p <0.001), respectively.”

In addition at pag. 7 Fig. 5 entitled “Changes in mean duration of inactivity/p. over 12 months” must be substituted with the new Fig. 5 because there is a mistake regarding **anova p = 0.11** and consequently in the whole image. This Correction shows the revised Fig. 5. The original article has been updated.

**Fig. 5:**
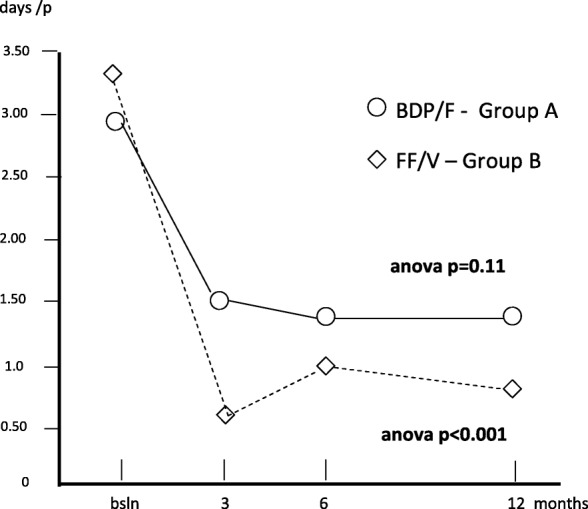
Changes in mean duration of inactivity/p. over 12 months
